# Genome Sequence of Enterotoxigenic *Escherichia coli* Strain FMU073332

**DOI:** 10.1128/genomeA.01600-16

**Published:** 2017-02-23

**Authors:** Zeus Saldaña-Ahuactzi, Ariadnna Cruz-Córdova, Gerardo E. Rodea, Helena Porta, Armando Navarro-Ocaña, Carlos Eslava-Campos, Miguel A. Cevallos, Juan Xicohtencatl-Cortes

**Affiliations:** a Laboratorio de Investigación en Bacteriología Intestinal, Hospital Infantil de México Federico Gómez, Ciudad de México, México; b Instituto de Biotecnología, Universidad Nacional Autónoma de México, Cuernavaca, Morelos, México; c Departamento de Salud Pública, Facultad de Medicina, Universidad Nacional Autónoma de México, Ciudad Universitaria, Ciudad de México, México; d Centro de Ciencias Genómicas, Programa de Genómica Evolutiva, Universidad Nacional Autónoma de México, Cuernavaca, Morelos, México

## Abstract

Enterotoxigenic *Escherichia coli* (ETEC) is an important cause of bacterial diarrheal illness, affecting practically every population worldwide, and was estimated to cause 120,800 deaths in 2010. Here, we report the genome sequence of ETEC strain FMU073332, isolated from a 25-month-old girl from Tlaltizapán, Morelos, México.

## GENOME ANNOUNCEMENT

Enterotoxigenic *Escherichia coli* (ETEC) is a type of pathogenic bacteria that causes traveler’s diarrhea and affect populations worldwide, with children under 2 years old being the most affected (1, 2). The mortality caused by ETEC in all age groups in 2010 was estimated to be 120,800 deaths (3). ETEC strain FMU073332 serotype O6:H16 is a clinical isolate, recovered in 1987 from the stool of a 25-month-old girl from Tlaltizapán, Morelos, México (4). This strain has been used in previous studies to characterize the CS21 colonization factor in the adherence of ETEC to HT-29 cells and self-aggregation (5). We used the genomic sequences of ETEC strain FMU073332 to extract gene sequences and perform multilocus sequence typing (MLST). According to the PubMLST (Achtman) and Pasteur systems, the strain belongs to the sequence type 4 (ST4) and ST215 clonal groups, respectively (6, 7). This strain carries the classic virulence genes *eltA*, *eltB*, *sta*2, *cstH*, and *lngA* and the nonclassic *etpA* and *etpB* genes.

A high-quality draft of the genome of this strain was determined on a PacBio RSII platform. A large-insert (20 to 25 kb) library was constructed and sequenced, using one single-molecule real-time (SMRT) cell with a P6 polymerase and C4 chemistry combination (P6-C4) with a 180-min movie. The statistical analysis obtained from the genome assembly gave 43,603 reads with an *N*_50_ length of 11,395 bp. The *de novo* assembly was made using an RS Hierarchical Genome Assembly Process (HGAP) protocol version 3 in SMRT Analysis version 2.3 (Pacific Biosciences) (8). To improve regions of low coverage, a library with a 2 × 100-bp paired-end configuration was sequenced on an Illumina HiSeq platform. This platform yielded 11,282,773 reads. A hybrid assembly was constructed with the Illumina HiSeq reads and PacBio RII subreads, using SPAdes 3.5.0 (9). The plasmids and chromosome were circularized, when possible, using apc, a perl script available at https://github.com/jfass/apc. Functional annotation was performed with the NCBI Prokaryotic Genome Annotation Pipeline.

The hybrid assembly consisted of five contigs: the largest comprises the complete ETEC strain FMU073332 circular chromosome (4,718,719 bp). The other four contigs represent the four plasmids of this strain: pEcoFMU073332a (5,538 bp), pEcoFMU073332b (47,563 bp), pEcoFMU073332c (113,343 bp), and pEcoFMU073332d (137,665 bp). The sequences of plasmids pEcoFMU073332b and pEcoFMU073332d are not complete; however, an electrophoretic plasmid profile analysis indicated that the number and sizes of the observed plasmids match with the sequence sizes of plasmids pEcoFMU073332b and pEcoFMU073332d from genome assembly.

The chromosome has seven rRNA operons, 91 tRNAs, and 4,936 coding sequences (CDSs). Some genes relevant for virulence are located in plasmids, for example, genes encoding subunits A and B of the heat-labile enterotoxin (*eltA* and *eltB*); the secreted autotransporter serine protease (*eatA*); and the mayor fimbrial subunit (*cstH*) and the heat-stable enterotoxin ST (*sta*2) located in plasmid pEcoFMU073332d. In contrast, gene encoding the CS21 pilus structural subunit (*lngA*) is located in plasmid pEcoFMU073332b. The high-quality draft genome of ETEC Mexican strain FMU073332 and its four plasmids will aid in precise genetic manipulation and therefore further improve the study of ETEC virulence.

### Accession number(s).

The complete sequences of the chromosome and the four plasmids of ETEC strain FMU073332 have been submitted to GenBank under the accession numbers CP017844 to CP017848.
